# Satellite‐Based Monitoring of Eutrophication in the Earth's Largest Transboundary Lake

**DOI:** 10.1029/2022GH000770

**Published:** 2023-04-29

**Authors:** Zohra Mozafari, Roohollah Noori, Seyed Mostafa Siadatmousavi, Hossein Afzalimehr, Jafar Azizpour

**Affiliations:** ^1^ School of Civil Engineering Iran University of Science and Technology Tehran Iran; ^2^ Graduate Faculty of Environment University of Tehran Tehran Iran; ^3^ Faculty of Governance University of Tehran Tehran Iran; ^4^ Iranian National Institute for Oceanography and Atmospheric Science (INIOAS) Tehran Iran

## Abstract

The world's large lakes and their life‐supporting services are rapidly threatened by eutrophication in the warming climate during the Anthropocene. Here, MODIS‐Aqua level 3 chlorophyll‐*a* data (2018–2021) were used to monitor trophic state in our planet's largest lake, that is, the Caspian Sea that accounts for approximately 40% of the total lacustrine waters on Earth. We also used the in situ measurements of chlorophyll‐*a* data (2009–2019) to further verify the accuracy of the data derived from the MODIS‐Aqua and to explore the deep chlorophyll‐*a* maxima (DCMs) in the south Caspian Sea. Our findings show an acceptable agreement between the chlorophyll‐*a* data derived from the MODIS‐Aqua and those measured in situ in the coast of Iran (coefficient of determination = 0.71). The oligotrophic, mesotrophic, and eutrophic states cover 66%, 20%, and 13% of the sea surface area, respectively. The DCMs are dominantly regulated by water transparency and they generally observe at depths of less than 20 and 30 m during the cold (autumn and winter) and warm (spring and summer) seasons, respectively. Our results suggest an ever‐increasing chlorophyll‐*a* in the shallow zones (i.e., coasts) and even in deep regions of the sea, mainly due to nutrient inputs from the Volga river delta. Alarming increase of chlorophyll‐*a* in this transboundary lake can amplify eutrophication under the lens of global warming and further threaten the lake ecosystem's health, where almost all legal agreements have not yet been implemented to protect the lake environment and its rich resources.

## Key Points

- MODIS‐Aqua chlorophyll‐*a* data were used to monitor the trophic state in our planet's largest lake, that is, the Caspian Sea
- Our findings suggest an ever‐increasing chlorophyll‐*a* and eutrophic state in the lake, which can threaten the lake ecosystem's health
- Increase of chlorophyll‐*a* in the Caspian Sea can further amplify eutrophication under the lens of global warming

## 1 Introduction

Around 117 million lakes (>2,000 m^2^) that cover 3.7% of the Earth's land surface are supporting, among other things, ever‐increasing demands of growing global population, providing habitats for different species, and acting as sentinels of environmental changes (Jenny et al., 2020; Verpoorter et al., 2014). They, however, are threatened by eutrophication: “a scientific terminology that defines algal blooms and associated consequences induced by the response of aquatic ecosystems to large nutrient loads” (Jenny et al., 2020; Schindler, 2001). Eutrophication makes changes in dissolved oxygen, intensifies fish death and growth of phytoplankton organisms, and produces toxins and bloom cyanobacteria, with acute implications for lake water quality, biodiversity, socio‐economic benefits, and recreational and tourism opportunities (Kim et al., 2021; Noori, Ansari, Jeong, et al., 2021; Schindler, 2001). Despite undeniable importance of lakes and key ecosystem services they provide (Jenny et al., 2020; Noori et al., 2022), a comprehensive understanding of the extent of eutrophication in large and deep lakes is relatively lacking, mostly due to, among other things, technical complications or/and scarce data to cover the large geographical region. Meanwhile, restoration of lakes and their life‐supporting services require an understanding of the eutrophication dynamics under frequent monitoring of the phytoplankton community (Jenny et al., 2020).

Phytoplankton groups, as the foundation of aquatic food chains, are microscopic organisms that live under presence of carbon dioxide, sunlight, and nutrients in both fresh and saline water ecosystems with photosynthetic capacity (Lindsey & Scott, 2010). Chlorophyll‐*a* (Chl‐*a*) is the most important photosynthetic pigment in phytoplankton organisms, which has been considered as a good indicator of the nutrients' enrichment (Agwanda & Iqbal, 2019; Kakore et al., 2022). Therefore, Chl‐*a* can be used as a biomass indicator of the trophic status in lakes (Atique & An, 2020; Koponen et al., 2001). The conventional method for estimating the Chl‐*a* concentration is to use data collected during field and laboratory studies (Aranha et al., 2022; Shoaib et al., 2019). However, field and laboratory methods are expensive and time consuming, especially in large lakes, where the difficulties raise up exponentially (Gholizadeh et al., 2016). In this context, remote sensing‐based technology has the potential to present a comprehensive estimate of the Chl‐*a* in lakes. Optically active components of water that interact with light and change the energy spectrum of solar radiation reflected from water can be measured using remote sensing (Ritchie et al., 2003). In this regard, different sensors onboard on satellites have been sophisticated to estimate the Chl‐*a*, namely the Coastal Zone Color Scanner Experiment (CZCS) (Gordon et al., 1983), Operational Land Imager (OLI) in Landsat (Irons et al., 2012), the Moderate Resolution Imaging Spectroradiometer (MODIS) (Justice et al., 2002), Sea‐Viewing Wide Field‐of‐View Sensor (SeaWiFS) in OrbView‐2 (Hooker et al., 1992), and Multi‐Spectral Instrument (MSI) in Sentinel‐2 (Toming et al., 2016). The CZCS started in 1978 and ended in 1986, MODIS has been in operation since 2002, SeaWiFs started in 1997 and ended in 2010, and MSI and OLI have been in operation since 2015 and 2013, respectively. These sensors measure the Chl‐*a* at, approximately, daily to bi‐weekly time intervals with a spatial resolution up to 10 m.

The Chl‐*a* data produced by MODIS instrument, onboard in both Terra and Aqua spacecraft, with an acceptable temporal resolution and spatial coverage can be considered as a good alternative to conventional methods for Chl‐*a* monitoring to study eutrophication in large lakes (Li et al., 2017; Wang et al., 2018). Here, we aim to investigate the recent spatial‐temporal changes in the Chl‐*a* concentration using the MODIS‐Aqua level 3 satellite data in our planet's largest lake, that is, the Caspian Sea, from 2018 to 2021. The Caspian Sea, which accounts for around 40% of the total lacustrine waters on Earth (https://web.archive.org/web/20090122212158/http://irangazette.com/12.html), becomes distinct from other large lakes by its special natural conditions, rich natural resources, diverse biodiversity, and important geopolitical‐economical role for its littoral states (Kostianoy & Kosarev, 2005). In this study, we also use the in situ measurements of Chl‐*a* (2009–2019) to verify the accuracy of the data derived from the MODIS‐Aqua and to explore the deep Chl‐*a* maxima (DCMs) in the south Caspian Sea. Our findings suggest an ever‐increasing Chl‐*a* in the shallow zones (i.e., coasts) and even in deep regions of the Caspian Sea, which can amplify eutrophication under the lens of global warming and further threaten the ecosystem health in this transboundary lake.

## 2 Materials and Methods

### 2.1 Study Area

**Figure 1:**
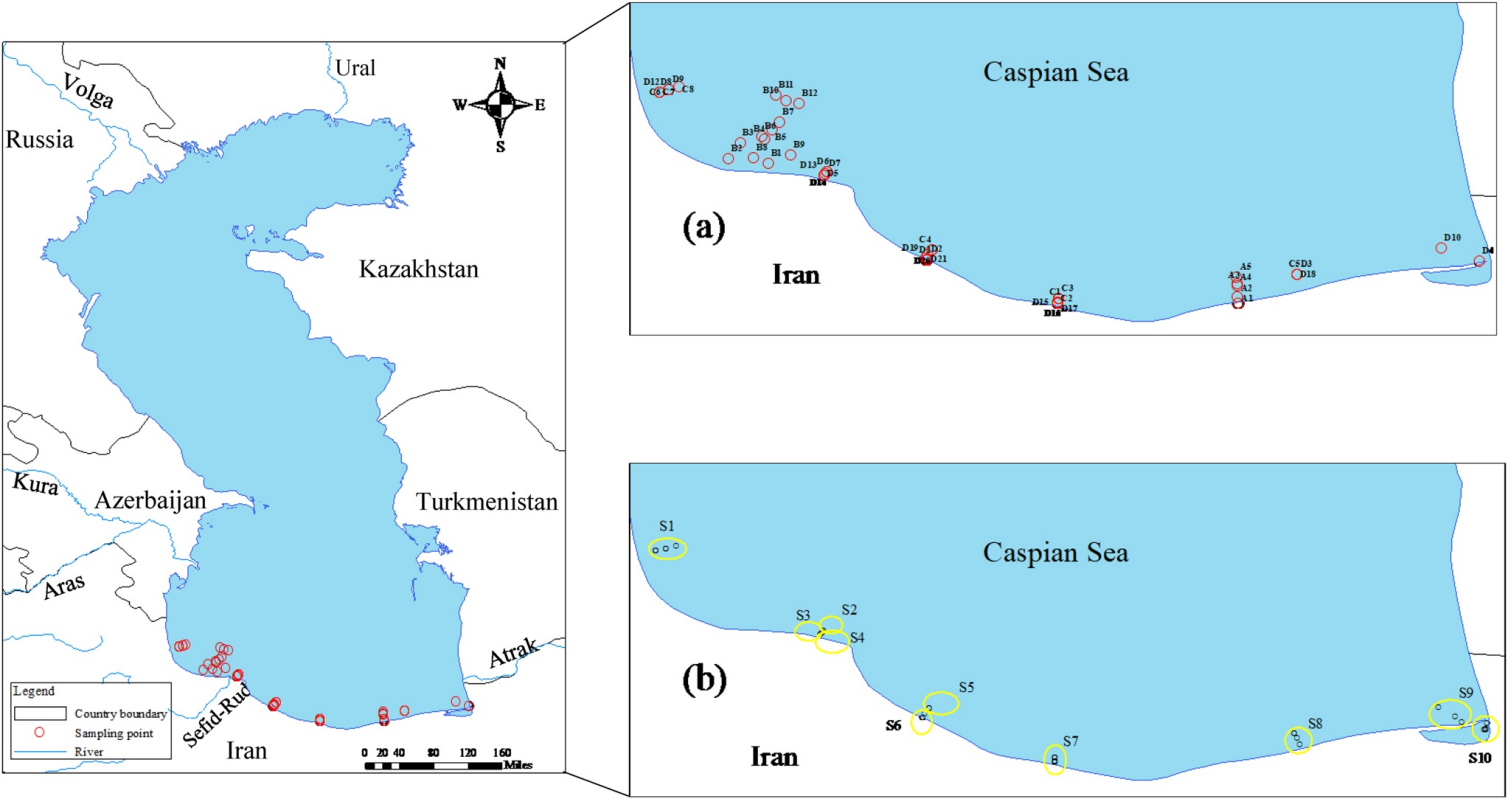
Caspian Sea and chlorophyll‐*a* (Chl‐*a*) monitoring stations across the coast of Iran. (a) The in situ measured Chl‐*a* data in red circular‐shaped stations were used to verify those ones estimated by the Moderate Resolution Imaging Spectroradiometer (MODIS) sensor onboard on the Aqua spacecraft (MODIS‐Aqua). A1–A5: Chl‐*a* data measured in 2009, B1–B12: Chl‐*a* data measured in 2012, C1–C8: Chl‐*a* data measured in 2018, and D1‐D21: Chl‐*a* data measured in 2019. (b) The in situ measured Chl‐*a* in black circular‐shaped stations were used for determination of the deep chlorophyll‐*a* maxima (DCMs). Due to non‐significant variation in Chl‐*a* measured at some adjacent sampling points, we merged these adjacent stations to distinct zones S1–S10 (yellow circular‐shaped zones S1–S10 in the panel (b)) for investigation of the DCMs in the south Caspian Sea.

The Caspian Sea is the largest lake by volume on Earth, bisected by the Asia‐Europe boundary. Kazakhstan, Azerbaijan, Russia, Turkmenistan, and Iran are the five littoral states which partake the coastline of the Caspian Sea (around 7,500 km), while Armenia, Georgia, Turkey, and Uzbekistan are non‐boarder countries in the endorheic basin of the sea (Figure 1). The sea's surface area is around 390,000 km^2^ (including the Garabogazköl lagoon to its east), which accounts for 40%–44% of the total lacustrine waters on Earth (https://web.archive.org/web/20090122212158/http://irangazette.com/12.html). The Caspian Sea prolongs around 1,200 km through north to south, with a mean width of 320 km. The sea with a water volume of 78,200 km^3^ is ∼1.7 times larger by volume than the five Laurentian Great Lakes and Lake Baikal pooled. Water level in the Caspian Sea is ∼27 m below the level of the high seas (Ghayebzadeh et al., 2020a; Kostianoy & Kosarev, 2005). The sea's maximum and mean depths are ∼1,025 and ∼184 m, respectively (Aladin & Plotnikov, 2004). According to the physical geographical conditions, the Caspian Sea can be divided into three parts: north, middle and south, which consist 29% (1%), 36% (35%), and 35% (64%) of the sea area (volume), respectively (Aladin & Plotnikov, 2004; Kostianoy & Kosarev, 2005).

Annual average air temperature on the sea varies from 10°C in the northern part to 17°C in the southern part. Sea surface temperature gets warmer from north to south in the range of 0°C–26°C (Dumont, 1998; Modabberi et al., 2020). About 130 rivers join to the Caspian Sea, where more than 90% of inflow to the sea is from northern and western fresh rivers, that is, Volga, Ural, Terek, Sulak, and Kura rivers. Among these, the Volga river, as the European largest river, is the main source of freshwater that enters the Caspian Sea (around 80% of inflow) at the shallow north end (Jamshidi, 2014; Mamedov, 1997; Rodionov, 1994). Because the sea is mainly nourishing with freshwater at the north, the water is almost fresh in its northern zone, gradually getting more brackish toward the south. The sea is mostly saline on the Iranian coast (excluding the Garabogazköl bay), where little flow enters the sea (Moradi, 2014).

The Caspian Sea is more vulnerable than the open seas to natural and human‐made pollution loads, due to its landlocked environment (Ghayebzadeh et al., 2020b). This sea was exposed to more anthropogenic pollution loads during the Soviet Union era, which negatively influenced the sea ecosystem. Following the dissolution of the Soviet Union, the need for protection of the sea ecosystem and its rich resources was aroused, led to signature of a multilateral agreement titled: “*Framework Convention for the Protection of the Marine Environment of the Caspian Sea*” or simply the “*Tehran Convention*” by the post‐Soviet countries in 4 November 2003 (https://tehranconvention.org/). However, almost all of the articles of this convention have not yet been entered into force by littoral states. Still, the development of urbanization and the increase of industrial and agricultural activities in the Caspian Sea watershed are unsustainable, leading to discharge of large pollution loads into the sea, mostly from the north end and the west coasts. During a long‐term monitoring program from 1978 to 2004, Korshenko and Gul (2006) reported high concentration of nutrients in the north Caspian Sea, which mainly discharged through the Volga river (Aladin & Plotnikov, 2004). This river basin alone contributes up to 50% of annual wastewater inputs to the Caspian Sea (23–25 km^3^) (Kostianoy & Kosarev, 2005). Ever‐increasing algal bloom events and other environmental hazards have been repeatedly reported in different parts of the Caspian Sea during the last two decades (Ivanov et al., 2012; Kopelevich et al., 2008; Korshenko & Gul, 2006; Moradi, 2013, 2014; Nasrollahzadeh et al., 2011; Roohi et al., 2021; Soloviev, 2005; Zonn, 2005). In this context, we aim to highlight the recent spatial‐temporal changes in Chl‐*a* concentration and the sea eutrophication using the MODIS‐Aqua level 3 satellite data in the Caspian Sea, from 2018 to 2021.

### 2.2 Data Collection

#### 2.2.1 Field Data

**Figure 2:**
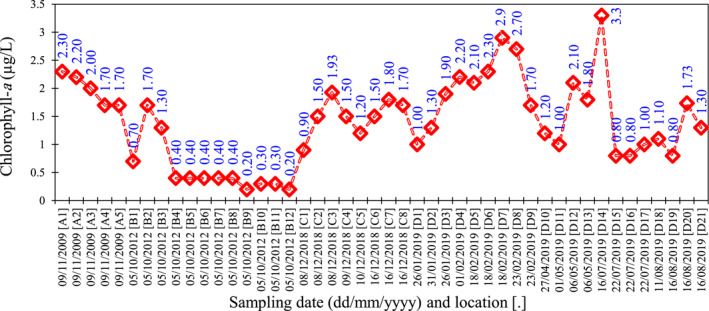
In situ measured chlorophyll‐*a* (Chl‐*a*) concentration in the south Caspian Sea (near the coast of Iran).

The in situ measured Chl‐*a* data were collected by the Iranian National Institute for Oceanography and Atmospheric Science (INIOAS). Chlorophyll‐*a* data were measured by the OCEAN SEVEN 316Plus CTD multi‐parameter probe manufactured by IDRONAUT Company, Italy (https://www.idronaut.it/). Here, the CTD stands for three parameters of conductivity (*C*), temperature (*T*), and depth (*D*), respectively. The SEAPOINT fluoro‐meter installed on the CTD monitors the Chl‐*a* concentration (with an accuracy of 0.1 μg/L) by directly measuring the fluorescence emission from a certain sample of water. This database was collected near the coast of Iran in 2009, 2012, 2018, and 2019 (Table S1 in Supporting Information S1). The geographical location of the sampling stations is shown in Figure 1. Also, the Chl‐*a* concentrations during the sampling campaigns are shown in Figure 2.

#### 2.2.2 Satellite Data

The MODIS‐Aqua data products used in our study were acquired from the NASA Ocean Biology Processing Group (https://oceancolor.gsfc.nasa.gov). The Chl‐*a* product incorporates two well‐known algorithms, the band ratio OCx algorithm (O’Reilly et al., 1998) and the color index algorithm suggested by Hu et al. (2012). The former algorithm is a statistical equation (a fourth‐order polynomial equation) which connects remote sensing reflectances (RSR) to the Chl‐*a* concentration. The algorithm suggested by Hu et al. (2012) is a reflectance difference relationship which employs the difference between the RSR in the green color band and a linearly reference formed between RSR in the blue color and red color bands. The suggested algorithms by O’Reilly et al. (1998) and Hu et al. (2012) are used for Chl‐*a* retrievals more than 0.2 and less than 0.15 μg/L, respectively. Both algorithms are blended by a weighted method for Chl‐*a* retrievals between 0.15 and 0.2 μg/L. The output of both merged algorithms is the near‐surface Chl‐*a* concentration in μg/L, which is included as a part of the standard Level‐2 and the Level‐3 product suites. Here, the daily, monthly, seasonal, and annual Level‐3 standard mapped images produced by MODIS‐Aqua were used for the whole of the investigated period (2018–2021). These data are aggregated into 9‐km spatial square grids and are available online via: https://oceancolor.gsfc.nasa.gov/products.

In this study, the daily data were used only for validation of the MODIS‐Aqua products using the in situ measurements in the south Caspian Sea. However, the daily Chl‐*a* data archived in NASA website have considerable gaps in time and space, especially in the north Caspian Sea. Given considerable missing Chl‐*a* data, we did not reconstruct the gaps in time and space. Alternatively, we used the mean monthly, seasonal and annual data to examine the changes in Chl‐*a* concentration in the different regions of the Caspian Sea.

### 2.3 Calculation of the Carlson's Ttrophic State Index (TSI)

There are several indices to determine the trophic status of the lakes (Ferreira et al., 2011). Among them, the Trophic State Index (TSI) suggested by Carlson has been widely used (Carlson, 1977). This index considers algal biomass based on three water quality parameters, including Chl‐*a*, total phosphorus and the Secchi depth. In addition, Carlson's TSI can be calculated using only one of aforementioned parameters to picture out the trophic status in lakes. Carlson developed this method for situations where no reliable data exist for all three water quality parameters (Carlson, 1977). Here, Chl‐*a* concentration, as the most important indicator of eutrophication, was considered to evaluate the trophic status in the Caspian Sea during the study period. Having the Chl‐*a* data recorded by the MODIS‐Aqua, Carlson's TSI was computed by Equation 1.

(1) $$TSI\left(Chl-a\right)=10\times \left(6-\frac{2.04-0.68\times \ln\left(Chl-a\right)}{\ln\left(2\right)}\right)$$

Theoretically, the Carlson's TSI varies from 0 to *∞*. 0 < TSI(Chl‐*a*) < 40, 40 ≤ TSI(Chl‐*a*) < 50, 50 ≤ TSI(Chl‐*a*) < 70, and 70 ≥ TSI(Chl‐*a*) indicate the oligotrophic, mesotrophic, eutrophic, and hypereutrophic conditions in lakes, respectively. Also, 0 ≤ Chl‐*a* < 2.61, 2.61 ≤ Chl‐*a* < 7.3, 7.3 ≤ Chl‐*a* < 56, and 70 ≥ Chl‐*a* are responsible for 0 < TSI(Chl‐*a*) < 40, 40 ≤ TSI(Chl‐*a*) < 50, 50 ≤ TSI(Chl‐*a*) < 70, and 70 ≥ TSI(Chl‐*a*), respectively (Carlson & Simpson, 1996).

### 2.4 Deep Chlorophyll‐*a* Maxima (DCMs)

Deep chlorophyll maximum, as a common feature in stratified aquatic systems, mostly takes place in oligotrophic and mesotrophic states (Scofield et al., 2020). The DCM and its location are ecologically important, which determine the zone of primary production in the water column and influence the cycle of nutrients in the lakes and oceans (Jamart et al., 1977; Leach et al., 2018; Letelier et al., 2004). Many factors such as light availability, vertical distribution of nutrients, and the location of thermal gradients in the water column are often considered as the main abiotic drivers in the formation of the DCM depth in lakes (Abbott et al., 1984; Cullen, 2015; Durham & Stocker, 2012; Fee, 1976).

In our study, the depth profiles of Chl‐*a* data (up to 50 m) measured at sampling points during 2018 and 2019 were used to calculate the DCMs in the south Caspian Sea (black circular‐shaped stations in Figure 1b). These samples were collected with ∼1 m interval through water column at 18 sampling points in spring and summer, 17 sampling points in autumn, and 24 sampling points in winter. Our investigations revealed non‐significant variation in Chl‐*a* measured at some adjacent sampling points, mainly due to close proximity to each other. Therefore, we merged these sampling points, which resulted in reduction of the points to 8 in spring, 9 in summer and autumn and 10 in winter (yellow circular‐shaped zones S1–S10 in Figure 1b) (Table S2 in Supporting Information S1).

### 2.5 Statistical Measures

The statistical parameters used for evaluating the performance of the MODIS‐Aqua products were the root mean square error (RMSE) and the coefficient of determination (*R* ^2^), defined as:

(2) $$RMSE=\sqrt{\frac{\sum_{i=1}^{n}{\left(x_{i}-x_{i}^{\prime }\right)}^{2}}{n}}$$

(3) $$R^{2}=\frac{{\left(\sum \left(x_{i}-\bar{x}\right)\left(x_{i}^{\prime }-\bar{x^{\prime }}\right)\right)}^{2}}{\sum {\left(x_{i}-\bar{x}\right)}^{2}\sum {\left(x_{i}^{\prime }-\bar{x^{\prime }}\right)}^{2}}$$

where, $x_{i}$ and $x_{i}^{\prime }$ are in situ measurements of Chl‐*a* in the Caspian Sea and those recorded by the MODIS‐Aqua, respectively; $\bar{x}$ and $\bar{x^{\prime }}$ are average of in situ measurements of Chl‐*a* and those recorded by the MODIS‐Aqua, respectively; and *n* is the number of measurements.

RMSE and *R* ^2^ vary from 0 to *∞* and 0 to 1, respectively. RMSE and *R* ^2^ values equal 0 and 1, respectively, indicate a full agreement between the in situ measurements of Chl‐*a* in the Caspian Sea and those recorded by the MODIS‐Aqua (Noori et al., 2017; Saghafi et al., 2009).

## 3 Results and Discussion

### 3.1 Validation of Chlorophyll‐*a* Data Estimated by the MODIS‐Aqua

**Figure 3:**
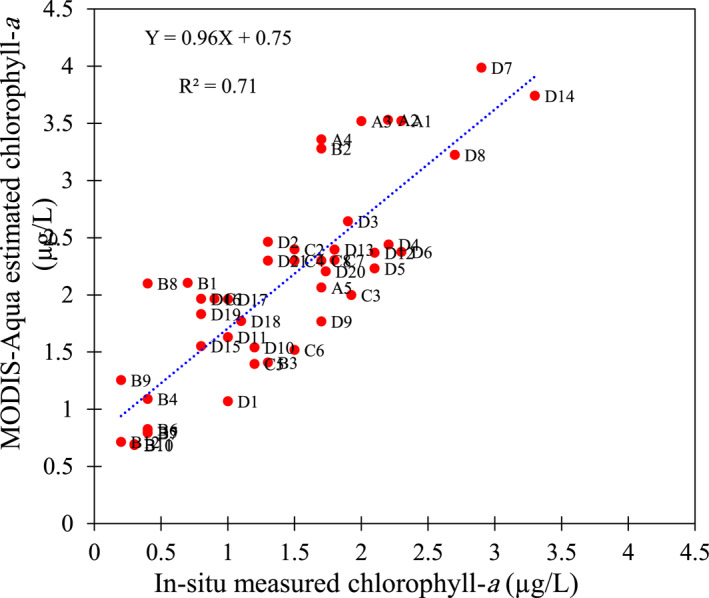
Scatter plot of the MODIS‐Aqua estimated chlorophyll‐*a* data versus those measured in situ in south Caspian Sea. A1–A5: The measured and the MODIS‐Aqua estimated Chl‐*a* data in 2009, B1–B12: The measured and the MODIS‐Aqua estimated Chl‐*a* data in 2012, C1–C8: The measured and the MODIS‐Aqua estimated Chl‐*a* data in 2018, and D1–D21: The measured and the MODIS‐Aqua estimated Chl‐*a* data in 2019. Geographical location of the sampling points A1–A5, B1–B12, C1–C8, and D1–D21 are shown in Figure 1a.

Given the temporal and spatial correspondence of the satellite‐based Chl‐*a* data with the in situ measurements and cloud‐free sky condition, 46 out of 99 in situ measured samples (red circular‐shaped stations in Figure 1a) were used for validation of the MODIS‐Aqua estimation of Chl‐*a* in the Caspian Sea. Our validation results suggest a good agreement between the in situ measured Chl‐*a* data and those estimated by the MODIS‐Aqua, with acceptable *R* ^2^ and RMSE values of 0.71 and 0.80, respectively. As shown in Figure 3, the satellite‐based Chl‐*a* data overestimate our in situ measurements in the south Caspian Sea. Different factors might contribute to such inconsistency between the Chl‐*a* estimated by the MODIS‐Aqua and those measured in south Caspian Sea. MODIS‐Aqua offers the gridded Chl‐*a* data with a 9‐km resolution. This means the satellite‐based Chl‐*a* data are smoothed in the coastal zones of the south Caspian Sea, where a sharp descent gradient of Chl‐*a* is usually observed toward the sea. Therefore, relatively large differences may exist between the MODIS‐Aqua Chl‐*a* data and those measured in situ, especially the samples taken from the shallow regions in the littoral zone. In addition, the exact time of in situ data collection in this study might not be consistent with the local overpass time of the MODIS‐Aqua over the Caspian Sea (Crosson et al., 2012). In fact, in situ data should be collected about two to 3 hours after the satellites' overpass time to increase the accuracy of OceanColor satellite data (Bailey et al., 2000).

It should be noted that we excluded the samples taken from the Gorgan bay for the validation process due to the significant differences between the satellite‐based estimations of Chl‐*a* data and those measured in situ in the bay. According to our investigations and the field samples measured in the Gorgan bay, we found that the satellite‐based Chl‐*a* concentrations in this area are around five‐fold greater than the corresponding in situ measured Chl‐*a* data. Gorgan bay is a shallow water body, which connects to the Caspian Sea via two small mouths. Given the large water residence time of the Gorgan bay (>100 days) (Kheirabadi et al., 2018; Modabberi et al., 2020; Ranjbar & Hadjizadeh Zaker, 2018), the satellite‐based studies were reported the bay region as a hotspot associated with Chl‐*a* in the Caspian Sea with a monthly estimated Chl‐*a* concentration up to 85 μg/L (Modabberi et al., 2020). However, in situ measured Chl‐*a* concentrations reveal a large overestimation of the satellite‐based Chl‐*a* in this bay. For example, at the beginning of February 2019, the in situ measured Chl‐*a* concentration in the bay was 2.3 μg/L whilst the corresponding MODIS‐Aqua showed a six‐fold overestimation of Chl‐*a* in this region, that is, 14.9 μg/L. At the same time, in another region of the Gorgan bay, the in situ measured Chl‐*a* was 1.9 μg/L and the corresponding satellite‐based Chl‐*a* was 14.4 μg/L. In 9 April 2019, the in situ measured Chl‐*a* concentrations in the bay were up to five‐fold smaller than those monitored through the MODIS‐Aqua. In the same month, the satellite‐based Chl‐*a* in the southern part of the bay was 20.5 μg/L whilst the in situ data showed a Ch‐*a* concentration around 3.3 μg/L. Shallowness, large water residence time, and small area of the Gorgan bay (Kheirabadi et al., 2018; Modabberi et al., 2020; Ranjbar & Hadjizadeh Zaker, 2018) may contribute to large error in estimated Chl‐*a* through the MODIS‐Aqua compared with those measured in situ.

Noted that the in situ Chl‐*a* measurements are rare, and often confidential, in the Caspian Sea. Therefore, we spatially confined the validation process in south regions of the sea (near to coast of Iran), where we had a better access to the Chl‐*a* data. Given the good temporal coverage of the Chl‐*a* data (2009–2019), the conclusion made on the agreement between the derived Chl‐*a* data from the MODIS‐Aqua and those measured in situ would be acceptable.

### 3.2 Changes in the Deep Chlorophyll‐*a* Maxima (DCMs)

**Figure 4:**
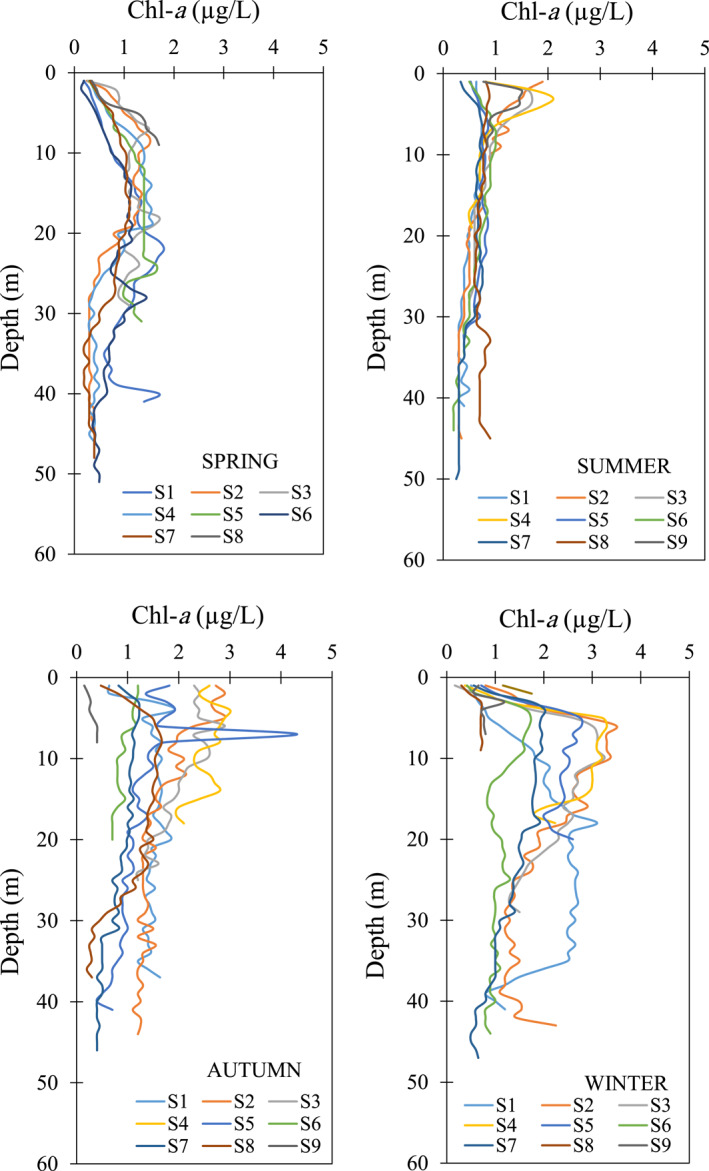
Seasonal variation of deep chlorophyll‐*a* maxima and their occurrence depth in the different parts of the south Caspian Sea. Geographical location of the sampling zones S1–S10 are shown in Figure 1b.

Depth variation of Chl‐*a* at different sampling zones is shown in Figure 4. According to this figure, the DCMs vary between 1.1 μg/L at the sampling zone S8 and 1.8 μg/L at the sampling zone S1 in spring. The depth of DCMs in spring starts from 7 m at the sampling zone S2 to 28 m at the sampling zone S7. In summer, the DCMs and their depths vary, respectively, from 0.77 (at S7) to 2.1 μg/L (at S4) and 1 (at S2) to 33 m (at S8). In autumn, the DCMs and their locations are within the range of 0.4 (at S9) to 4.3 μg/L (at S5) and 1 (at S2) to 17 m (at S6), respectively. In the winter season, the depth of DCMs varies from 2 m at the sampling zone S10 to 18 m at the sampling zone S1. The DCMs also vary from 0.7 μg/L at the sampling zone S8 to 3.5 μg/L at the sampling zone S2 during the winter season. In general, the DCMs occurred at a lower depth in the sampling zone S2 and S9 due to close proximity to the shallow coasts.

The maximum DCM values observed in autumn and winter during the study period when the south Caspian Sea was impacted by the invasion of Jellyfish species named as “M. leidy” (Roohi et al., 2021). Kideys et al. (2008) investigated the impact of “M. leidy” invasion on the seasonal changes of Chl‐*a* concentration in the south Caspian Sea (Kideys et al., 2008). They concluded a significant relationship between the high levels of chlorophyll and M. leidy invasion that occurred in winter. In general, after this invasion, an increase in Chl‐*a* concentration about 5 μg/L was observed in winter (Moradi, 2013). However, the average depth of DCM in the south Caspian Sea is 7.8 m during summer, which is close to the depth of DCM averaged in 100 lakes around the world in summer season (i.e., 9.2 m) (Leach et al., 2018).

It should be noted that the average depth of DCM in the south Caspian Sea is still shallower than that reported for the global lakes. DCM investigation in the 100 global largest lakes showed that light attenuation was more important than thermal stratification in predicting the DCM depth, suggesting the DCM becomes deeper with increasing the lake transparency (Leach et al., 2018). Also, the thickness of the DCM increases as the lake becomes larger. In addition, the results showed that the relative importance of light and heat in the structure of DCM was not uniform among the different types of lakes (Leach et al., 2018). In previous studies, the thermocline depth in the south Caspian Sea was reported between 20 and 50 m (Jamshidi, 2017). However, our results show the depth of DCM is usually shallower than the depth of thermocline in the south Caspian Sea. In our study, the samples were collected from the south Caspian Sea, where Chl‐*a* concentration is usually higher than that of in the central Caspian Sea. Higher levels of Chl‐*a* further prevent light penetration in the water column, leading to dominant impact of light compared to the heat on the DCM. Therefore, the light penetration further regulates the depth of DCM in the south Caspian Sea compared to the thermocline depth.

### 3.3 Spatio‐Temporal Changes in Chlorophyll‐*a* Concentration

**Figure 5:**
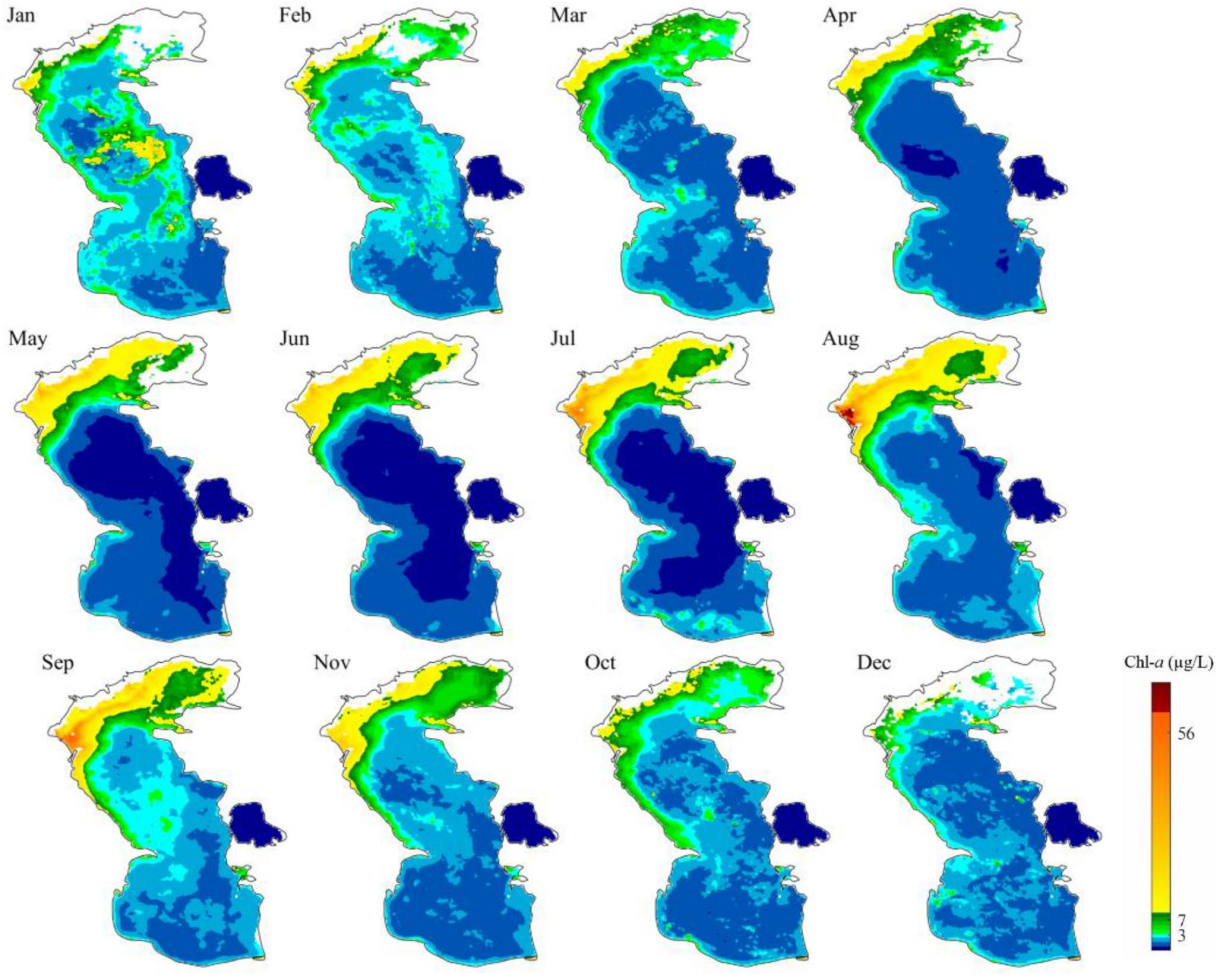
Spatial distribution of monthly mean concentration of chlorophyll‐*a* (Chl‐*a*) in the Caspian Sea from 2018 to 2021.

**Figure 6:**
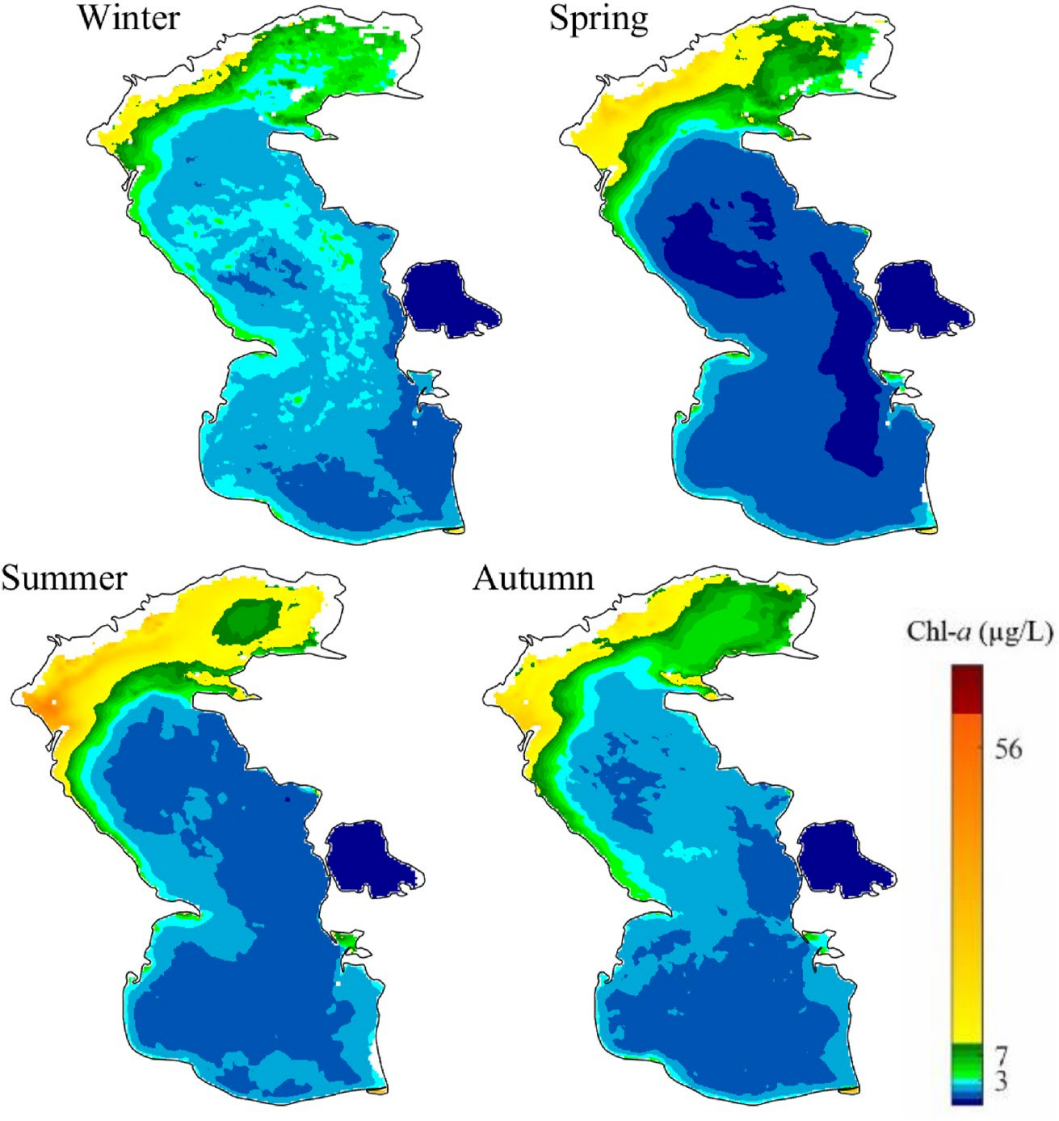
Spatial distribution of seasonal mean concentration of chlorophyll‐*a* (Chl‐*a*) in the Caspian Sea from 2018 to 2021.

**Figure 7:**
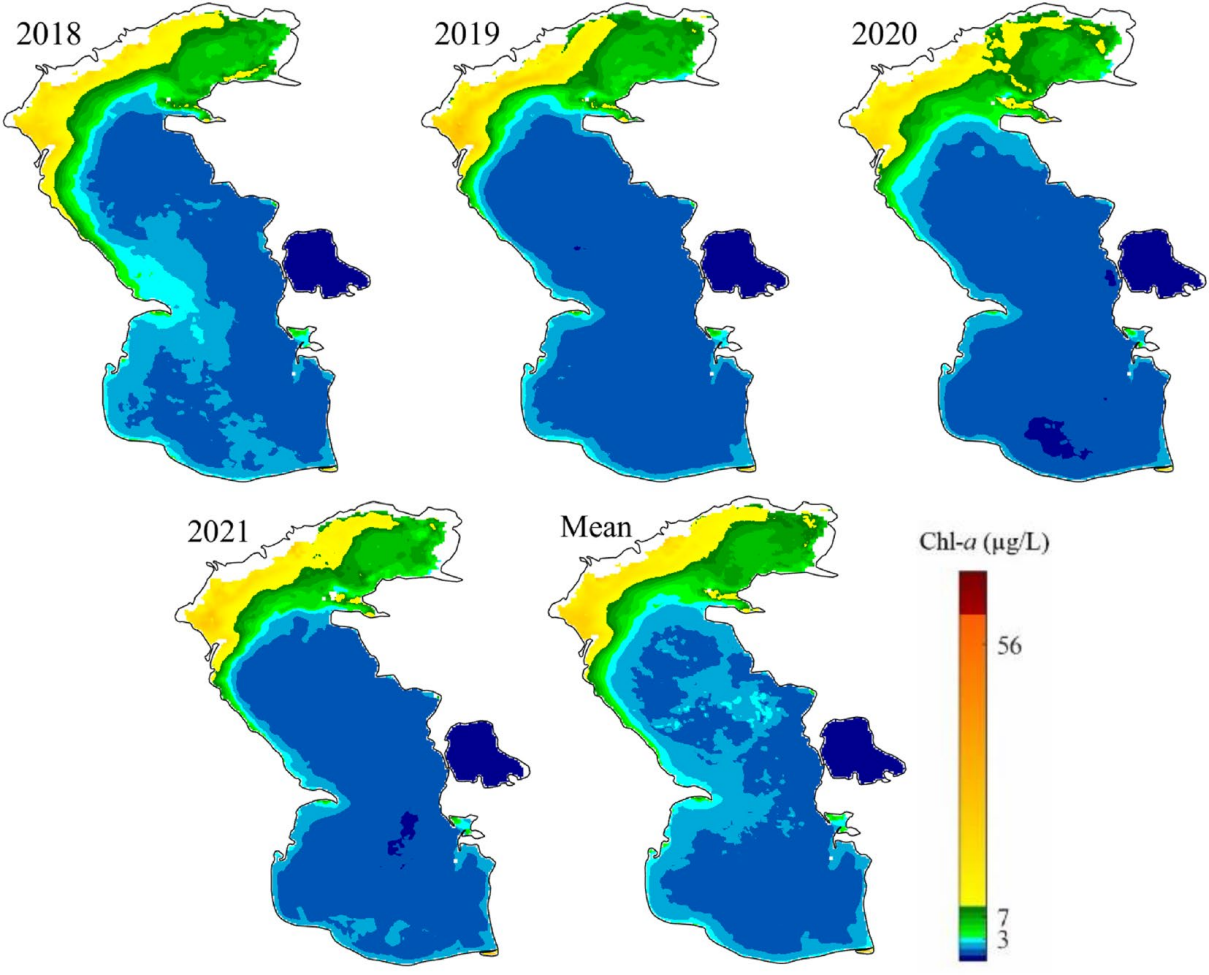
Spatial distribution of annual mean chlorophyll‐*a* (Chl‐*a*) in years of 2018–2021, and mean annual Chl‐*a* in the Caspian Sea during the study period (2018–2021).

The monthly, seasonal, and annual average of Chl‐*a* concentration in the Caspian Sea was calculated both temporally and spatially during the study period. The north Caspian Sea, especially the parts close to the coast of Russia, for example, the delta of Volga river, are exposed to high concentrations of Chl‐*a* in monthly, seasonal and annual time scales during the study period (Figures 5, 6, 7). The Volga river basin is the home of ∼40% of the Russia population and contains ∼45% of the country's agricultural and industrial activities (Schletterer et al., 2019). This river basin contributes to around 30%–35% of the total events of high to extremely pollution of surface waters in the vast country of Russia (Polianin & Kirpichnikova, 2020) and brings more than 80% of the organic and biogenic compounds to the Caspian Sea (Shiganova et al., 2003). Published scholarly works have shown that around 91% and 86% of the discharged nitrogen and phosphorus compounds, respectively, come from the coast of Russia (Stol'berg et al., 2006). In addition to the north, western region of the Caspian Sea also shows high concentrations of Chl‐*a*, where the Terek, Samur, Sulak, and Kura rivers discharge large nutrient loads to the sea (Nasrollahzadeh et al., 2008).

Figure 5 shows that the Chl‐*a* concentration has increased in the Volga river delta from April to October. Recent studies have also highlighted the increase in the nutrient loads originating from (non)point sources of pollution in the Volga river basin, especially during spring flood events and agricultural season (Polianin & Kirpichnikova, 2020). The intense discharge of nutrients from the northern rivers to the sea leads to an increase in the concentration of Chl‐*a* in the north coast of the Caspian Sea, contributing to a rise up of Chl‐*a* in the middle and south regions with a lag time of up to 4 months (Moradi, 2022). The surface currents produced in the north of the Caspian Sea move southward across the west shorelines and mainly spread large nutrient loads as the main contributor of Chl‐*a* in the sea (Diansky et al., 2018; Kostianoy & Kosarev, 2005).

High concentrations of Chl‐*a* are also observed on the shores of Baku in Azerbaijan, as well as in the coastal parts of Kazakhstan during the study period, but they are still lower than those observed in the shores of Russia. Satellite data show high concentrations of Chl‐*a* in the Gorgan bay, located in the Iranian side of the Caspian Sea. As discussed before, the satellite data are not consistent with the in situ measured Chl‐*a* data in the bay. Although the southern and, especially, the middle parts of the Caspian Sea generally have lower Chl‐*a* concentration than the northern part, high concentrations of Chl‐*a* are observed in these regions during January and February (winter months) (Figure 5). This can be attributed to the downward transport of cold nutrient‐rich currents along the northwest shelf, originated from the discharge of northern rivers into the Caspian Sea and its counterclockwise circulation. After reaching the Apsheron Peninsula in Azerbaijan, the cold nutrient‐rich water flows eastward and traps in strong eddies in deep regions of the middle Caspian Sea (Sur et al., 2000). This dominant hydrodynamic feature in the Caspian Sea could contribute to higher Chl‐*a* concentration in west than in east coasts, as well as move the algae to the middle and south regions of the sea.

The peak of monthly mean Chl‐*a* concentration (up to 90 μg/L) occurred in August, followed by September (up to 60 μg/L) in the north Caspian Sea (Figure 5). In addition to the discharge of large pollution loads to the sea during the agricultural season (Polianin & Kirpichnikova, 2020), the maximum sea surface temperature, intensive solar radiation, and wind stress may also contribute to the peak of Chl‐*a* in August and September in this region (Nezlin, 2006). This finding is also in line with the historical record of an intensive algal bloom occurred in the south Caspian Sea during August 2005, a phenomenon that covered an area of 20,000 km^2^ (Soloviev, 2005), and the algal blooms happen in the warm season of 2007, 2009, and 2010 (Nasrollahzadeh et al., 2011).

The north Caspian Sea is less impacted by the extreme high concentration of Chl‐*a* in the first quarter of each year, that is, from February to March (less than 32 μg/L) (Figure 6). The extreme high concentration of Chl‐*a* is also observed in the north Caspian Sea during summer, which can be due to the nutrient inputs and provision of suitable environmental conditions such as light and temperature for the growth of algae, as both discussed before. However, the south and middle regions of the Caspian Sea experience higher concentration of Chl‐*a* during winter, which is in‐line with the field studies conducted by Makhlough et al. (2021) during the year of 2018–2019 period. The pattern of seasonal and annual change of Chl‐*a* concentration is very similar to each other, and both are like the monthly pattern.

### 3.4 Trophic State Over the Caspian Sea

**Figure 8:**
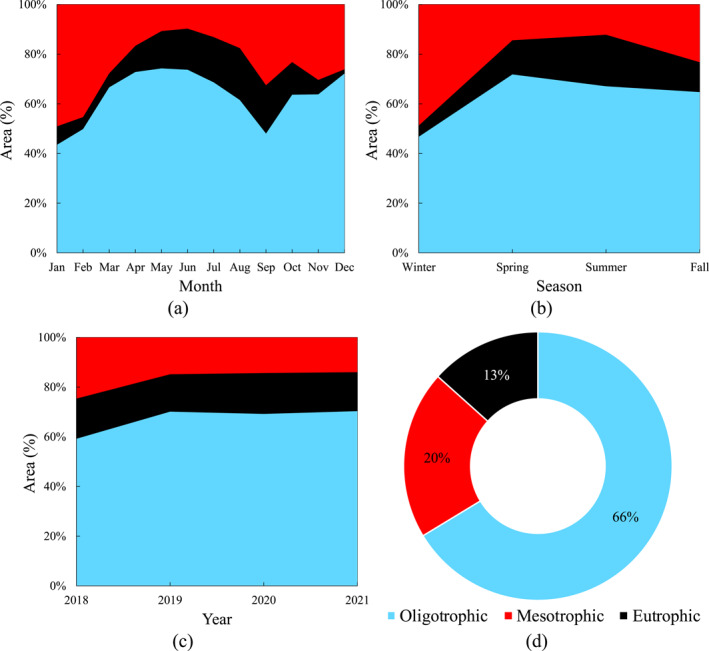
Area percentage of the Caspian Sea covered by oligotrophic (light blue color), mesotrophic (red color), and eutrophic (black color) conditions in the different time scales: (a) monthly, (b) seasonal, (c) annual, and (d) whole study period (average of monthly values from 2018 to 2021).

Using the monthly mean Chl‐*a* obtained from the MODIS‐Aqua products, the spatial distribution maps of TSI in the Caspian Sea were calculated separately for monthly, seasonal, and annual time scales during the study period (2018–2021). Based on the calculated TSI, the percentage of sea regions subjected to oligotrophic, mesotrophic, and eutrophic conditions was calculated for different time scales (Figure 8).

The temporal variation of monthly mean TSI shows that approximately 21%, 19%, and 18% of the sea zone are in the worst trophic conditions, that is, eutrophic state, during August, September, and July, respectively. The best trophic condition is observed in April, June, and May, and December, when oligotrophic waters cover up to 70% of the sea zone. Mesotrophic state is the dominant trophic class in the Caspian Sea during January (49% of the sea zone) and February (45% of the sea zone) (Figure 8a). Around 5%, 14%, 21%, and 12% of the sea regions are eutrophic during winter, spring, summer, and autumn, respectively. These findings reveal that more area of the sea is seasonally eutrophic compared with the previous results reported by Modabberi et al. (2020) from 2003 to 2017. More specifically, the sea zone covered by the eutrophic condition during summer season (i.e., 21%) from 2018 to 2021 is approximately 1.31 times greater than that of reported from 2003 to 2017 (i.e., 16%) (Modabberi et al., 2020) (Figure 8b). Other researchers also reported the same concerns about the alarming increase of eutrophication in the Caspian Sea during the recent years (Ahmadi et al., 2020; Makhlough et al., 2021; Moradi, 2014). This increasing trend of eutrophication can be intensified under the lens of global warming and further threaten the sea ecosystem's health (Gao et al., 2017; Lu et al., 2019; Noori, Ansari, Bhattari, et al., 2021), where almost all of legal agreements have not yet implemented to protect the lake environment and its rich resources. However, inter‐annual distributions of the TSI reveal that around 16% of the sea zone is eutrophic during the study period. Although some oligotrophic waters turned into mesotrophic state in 2018, no significant change was observed in the area percentage of the sea covered by oligotrophic, mesotrophic, and eutrophic waters from 2019 to 2021 (Figure 8c). Annual mean areas of the sea covered by oligotrophic, mesotrophic, and eutrophic waters are 66%, 20%, and 13%, respectively (Figure 8d).

## 4 Conclusion

In this study, we investigated the recent spatial‐temporal changes in Chl‐*a* concentration using the MODIS‐Aqua data in the Caspian Sea (2018–2021) to highlight the trophic conditions which can threaten the sea ecosystem's health. We also used Chl‐*a* data from several CTD casts to explore the accuracy of the satellite‐based Chl‐*a* data and the DCMs in the south Caspian Sea. Our findings suggested the north Caspian Sea, especially the northwest end, experienced higher Chl‐*a* concentration compared to the south and middle regions, mainly due to large nutrient load inputs from the Volga river. Carlson's TSI showed that eutrophic waters seasonally cover more area of the sea, especially during summer, compared with those covered 2003–2017. Our analysis also revealed the satellite‐based Chl‐*a* data matched with the in situ observations well. The depth of the DCM was mainly regulated by the light attenuation than the thermocline depth in the south Caspian Sea, in‐line with the results reported for the global large lakes.

As the eutrophic waters are expected to further cover the sea area in the future, the nutrient inputs to the sea should be regularly monitored by the littoral states. Also, we urge that further investigations are needed to improve our understanding of the sea response to the anthropogenic (e.g., unsustainable developments in the sea watershed) and natural (e.g., changes in local climate) drivers of eutrophication, which can threaten the sea ecosystem's health.

## Conflict of Interest

The authors declare no conflicts of interest relevant to this study.

## Supporting information

Supporting Information S1

## Data Availability

The raw data of Chl‐*a* are publicly available through https://oceancolor.gsfc.nasa.gov/products. The in situ measured Chl‐*a* data are given in Figures 1, 2, 3, 4. We have also provided the related processed Chl‐*a* data in Tables S1 and S2 in Supporting Information S1.
