# Supplementary material for: Satellite‐Based Monitoring of Eutrophication in the Earth's Largest Transboundary Lake
Source: Geohealth. 2023 Apr 29;7(5):e2022GH000770. doi: 10.1029/2022GH000770 (PMC10148676; doi:10.1029/2022GH000770)
Supplement: Supplementary file 1 — Supporting Information S1 [file GH2-7-e2022GH000770-s001.pdf]

[GeoHealth]

Supporting Information for

Satellite-Based Monitoring of Eutrophication in the Earth's Largest Transboundary Lake

Zohra Mozafari[1], Roohollah Noori[2,3,*], Seyed Mostafa Siadatmousavi[1], Hossein Afzalimehr[1], Jafar Azizpour[4]

[1]School of Civil Engineering, Iran University of Science and Technology, Narmak, Tehran 1684613114, Iran.

[2]Graduate Faculty of Environment, University of Tehran, Tehran, 1417853111, Iran.

[3]Faculty of Governance, University of Tehran, Tehran, 1439814151, Iran.

[4]Iranian National Institute for Oceanography and Atmospheric Science (INIOAS), Tehran, 1411813389, Iran.

Corresponding author: Roohollah Noori (noor@ut.ac.ir); ORCID: http://orcid.org/0000-0002-7463-8563

Contents of this file

Tables S1 and S2

Table S1. The *in-situ* measured Chl-*a* data (µg/L) which collected by the Iranian National Institute for Oceanography and Atmospheric Science (INIOAS) near the coast of Iran in 2009, 2012, 2018, and 2019. Geographical location of the sampling points is shown in the panel (a) of Fig. 1.

Station	In-situ measured Chl-*a* (µg/L)	Chl-*a* derived from the MODIS-Aqua (µg/L)	Sampling date	Station	In-situ measured Chl-*a* (µg/L)	Chl-*a* derived from the MODIS-Aqua (µg/L)	Sampling date
A1	2.30	3.52	09.11.2009	D1	1.00	1.07	26.01.2019
A2	2.20	3.53	09.11.2009	D2	1.30	2.46	31.01.2019
A3	2.00	3.52	09.11.2009	D3	1.90	2.64	26.01.2019
A4	1.70	3.36	09.11.2009	D4	2.20	2.44	01.02.2019
A5	1.70	2.07	09.11.2009	D5	2.10	2.23	18.02.2019
B1	0.70	2.10	05.10.2012	D6	2.30	2.38	18.02.2019
B2	1.70	3.28	05.10.2012	D7	2.90	3.99	18.02.2019
B3	1.30	1.41	05.10.2012	D8	2.70	3.22	23.02.2019
B4	0.40	1.09	05.10.2012	D9	1.70	1.77	23.02.2019
B5	0.40	0.80	05.10.2012	D10	1.20	1.54	27.04.2019
B6	0.40	0.83	05.10.2012	D11	1.00	1.63	01.05.2019
B7	0.40	0.79	05.10.2012	D12	2.10	2.37	06.05.2019
B8	0.40	2.10	05.10.2012	D13	1.80	2.40	06.05.2019
B9	0.20	1.26	05.10.2012	D14	3.30	3.74	16.07.2019
B10	0.30	0.69	05.10.2012	D15	0.80	1.55	22.07.2019
B11	0.30	0.69	05.10.2012	D16	0.80	1.97	22.07.2019
B12	0.20	0.72	05.10.2012	D17	1.00	1.96	22.07.2019
C1	0.90	1.97	08.12.2018	D18	1.10	1.77	11.08.2019
C2	1.50	2.40	08.12.2018	D19	0.80	1.83	16.08.2019
C3	1.93	2.00	08.12.2018	D20	1.73	2.21	16.08.2019
C4	1.50	2.30	09.12.2018	D21	1.30	2.30	16.08.2019
C5	1.20	1.40	10.12.2018				
C6	1.50	1.52	16.12.2018				
C7	1.80	2.31	16.12.2018				
C8	1.70	2.30	16.12.2018				

Table S2. Depth profiles of Chl-*a* data (up to 50 m) measured at the sampling zones during 2018 and 2019 in the south Caspian Sea. Geographical location of the sampling zones is shown in the panel (b) of Fig. 1.

WINTER											
No.	Water depth (m)	Depth profile of Chl-*a* (µg/L) in different sampling zones									
		S1	S2	S3	S4	S5	S6	S7	S8	S9	S10
1	0-1	0.50	0.80	0.17	0.38	0.71	0.42	0.55	0.30	0.65	1.16
2	1-2	0.57	1.30	0.60	0.80	1.03	0.61	0.97	0.47	0.57	1.75
3	2-3	0.65	1.57	1.20	1.20	1.55	1.23	1.73	0.70	1.18	
4	3-4	0.78	2.03	1.75	2.10	2.30	1.63	2.02	0.71	0.73	
5	4-5	0.87	2.90	2.60	3.20	2.75	1.73	1.95	0.70	0.77	
6	5-6	1.10	3.50	3.00	3.30	2.78	1.70	1.92	0.70	0.77	
7	6-7	1.32	3.30	3.10	3.20	2.60	1.62	1.97	0.70	0.80	
8	7-8	1.56	3.30	3.10	3.10	2.68	1.57	1.93	0.73		
9	8-9	1.77	3.30	3.10	3.10	2.40	1.60	1.97	0.70		
10	9-10	1.85	3.37	3.25	3.20	2.53	1.43	1.82			
11	10-11	2.12	3.00	2.90	3.00	2.50	1.27	1.82			
12	11-12	2.03	2.70	2.65	3.00	2.35	1.13	1.83			
13	12-13	2.00	2.70	2.70	3.00	2.35	0.93	1.83			
14	13-14	2.12	2.60	2.60	2.95	2.40	0.87	1.80			
15	14-15	2.17	2.70	2.60	2.70	2.43	0.83	1.77			
16	15-16	2.42	2.90	2.40	2.05	2.30	0.85	1.77			
17	16-17	2.61	2.50	2.60	1.80	2.00	0.97	1.80			
18	17-18	3.10	2.43	2.50	2.23	2.10	0.93	1.92			
19	18-19	2.59	1.90	2.30		2.20	0.98	1.59			
20	19-20	2.52	1.90	2.30		2.60	1.13	1.54			
21	20-21	2.60	1.90	2.10			1.15	1.48			
22	21-22	2.50	1.60	1.90			1.20	1.53			
23	22-23	2.55	1.77	1.70			1.15	1.58			
24	23-24	2.70	1.73	1.60			1.18	1.48			
25	24-25	2.65	1.40	1.50			1.30	1.38			
26	25-26	2.65	1.35	1.40			1.03	1.38			
27	26-27	2.68	1.40	1.30			0.98	1.35			
28	27-28	2.70	1.30	1.30			1.00	1.30			

Table S2. Depth profiles of Chl-*a* data (up to 50 m) measured at the sampling zones during 2018 and 2019 in the south Caspian Sea. Geographical location of the sampling zones is shown in the panel (b) of Fig. 1.

WINTER											
No.	Water depth (m)	Depth profile of Chl-*a* (µg/L) in different sampling zones									
		S1	S2	S3	S4	S5	S6	S7	S8	S9	S10
29	28-29	2.55	1.20	1.50			1.00	1.40			
30	29-30	2.65	1.30				0.98	1.10			
31	30-31	2.50	1.20				0.95	1.10			
32	31-32	2.50	1.25				1.00	1.00			
33	32-33	2.65	1.40				0.91	1.00			
34	33-34	2.50	1.30				1.05	1.00			
35	34-35	2.50	1.50				1.00	1.00			
36	35-36	2.00	1.30				1.10	1.00			
37	36-37	1.50	1.20				0.90	1.00			
38	37-38	1.20	1.20				0.90	0.90			
39	38-39	0.80	1.10				0.90	0.80			
40	39-40	1.00	1.49				1.00	0.80			
41	40-41	1.20	1.54				0.80	0.60			
42	41-42		1.42				0.80	0.60			
43	42-43		2.25				0.80	0.60			
44	43-44						0.90	0.50			
45	44-45							0.50			
46	45-46							0.60			
47	46-47							0.65			
SPRING											
No.	Water depth (m)	Depth profile of Chl-*a* (µg/L) in different sampling zones									
		S1	S2	S3	S4	S5	S6	S7	S8	S9	S10
1	0-1	0.19	0.25	0.27	0.73	0.31	0.30	0.20	0.32	0.35	
2	1-2	0.30	0.60	0.80	1.05	0.39	0.40	0.14	0.42	0.44	
3	2-3	0.35	0.75	0.90	1.00	0.46	0.50	0.25	0.50	0.52	
4	3-4	0.42	0.90	0.90	1.00	0.60	0.60	0.35	0.62	0.72	
5	4-5	0.48	1.00	1.10	1.10	0.67	0.70	0.43	0.77	1.25	
6	5-6	0.53	1.20	1.25	1.23	0.80	0.80	0.50	0.78	1.41	

Table S2. Depth profiles of Chl-*a* data (up to 50 m) measured at the sampling zones during 2018 and 2019 in the south Caspian Sea. Geographical location of the sampling zones is shown in the panel (b) of Fig. 1.

WINTER											
No.	Water depth (m)	Depth profile of Chl-*a* (µg/L) in different sampling zones									
		S1	S2	S3	S4	S5	S6	S7	S8	S9	S10
7	6-7	0.57	1.30	1.50	1.20	1.03	0.80	0.57	0.87	1.45	
8	7-8	0.63	1.50	1.30	0.90	1.20	1.00	0.63	0.92	1.63	
9	8-9	0.70	1.50	1.20	1.00	1.34	1.10	0.70	0.93	1.70	
10	9-10	0.77	1.30	1.10	1.10	1.40	1.20	0.75	1.03		
11	10-11	0.83	1.30	1.10	1.10	1.40	1.25	0.90	1.05		
12	11-12	0.98	1.30	1.10	1.10	1.33	1.40	1.00	1.03		
13	12-13	1.07	1.20	1.00	0.90	1.40	1.40	1.02	1.07		
14	13-14	1.20	1.20	1.15	1.00	1.55	1.40	1.13	1.03		
15	14-15	1.23	1.35	1.10	1.50	1.45	1.40	1.13	1.03		
16	15-16	1.34	1.30	1.30	2.60	1.45	1.40	1.10	1.12		
17	16-17	1.30	1.30	1.30	2.90	1.55	1.40	1.12	1.10		
18	17-18	1.27	1.20	1.70	3.35	1.50	1.40	1.07	1.10		
19	18-19	1.30	1.20	1.60		1.55	1.40	1.17	1.03		
20	19-20	1.45	0.80	1.30		0.90	1.40	1.10	1.03		
21	20-21	1.70	0.90	1.15		1.00	1.40	1.15	0.90		
22	21-22	1.80	0.70	1.00		1.00	1.40	0.90	0.90		
23	22-23	1.68	0.50	1.20		0.90	1.40	0.85	0.90		
24	23-24	1.60	0.50	1.30		0.78	1.65	0.75	0.85		
25	24-25	1.47	0.50	1.10		0.60	1.60	0.75	0.83		
26	25-26	1.23	0.40	1.00		0.50	1.10	0.95	0.80		
27	26-27	1.20	0.40	0.90		0.40	1.00	1.15	0.83		
28	27-28	1.20	0.30	0.90		0.35	1.00	1.45	0.78		
29	28-29	1.13	0.30	1.10		0.30	1.20	1.20	0.60		
30	29-30	1.00	0.30			0.40	1.20	1.00	0.50		
31	30-31	0.90	0.30			0.30	1.35	1.00	0.50		
32	31-32	0.80	0.30			0.30		0.80	0.35		
33	32-33	0.80	0.30			0.37		0.80	0.30		
34	33-34	0.70	0.30			0.45		0.70	0.20		

Table S2. Depth profiles of Chl-*a* data (up to 50 m) measured at the sampling zones during 2018 and 2019 in the south Caspian Sea. Geographical location of the sampling zones is shown in the panel (b) of Fig. 1.

**WINTER**											
No.	Water depth (m)	Depth profile of Chl-*a* (µg/L) in different sampling zones									
		S1	S2	S3	S4	S5	S6	S7	S8	S9	S10
35	34-35	0.60	0.30			0.40		0.70	0.20		
36	35-36	0.65	0.30			0.40		0.70	0.30		
37	36-37	0.75	0.30			0.47		0.60	0.20		
38	37-38	0.70	0.35			0.40		0.60	0.20		
39	38-39	0.90	0.30			0.50		0.64	0.20		
40	39-40	1.70	0.30			0.40		0.65	0.30		
41	40-41	1.40	0.30			0.40		0.50	0.30		
42	41-42		0.40			0.30		0.40	0.30		
43	42-43		0.35			0.40		0.40	0.30		
44	43-44		0.40			0.40		0.37	0.30		
45	44-45		0.40			0.30		0.42	0.40		
46	45-46					0.40		0.42	0.40		
47	46-47							0.50	0.40		
48	47-48							0.45	0.40		
49	48-49							0.40			
50	49-50							0.50			
51	50-51							0.50			
**SUMMER**											
No.	Water depth (m)	Depth profile of Chl-*a* (µg/L) in different sampling zones									
		S1	S2	S3	S4	S5	S6	S7	S8	S9	S10
1	0-1	0.63	1.90	0.80	0.80	0.53	0.50	0.33	0.82	0.77	1
2	1-2	0.63	1.60	1.60	1.70	0.58	0.60	0.40	0.87	1.48	2
3	2-3	0.67	1.50	1.70	2.10	0.65	0.70	0.53	0.88	1.45	3
4	3-4	0.70	1.25	1.65	1.90	0.70	0.75	0.67	0.83	1.43	4
5	4-5	0.70	1.10	1.40	1.50	0.80	0.90	0.70	0.83	1.00	5
6	5-6	0.73	1.05	1.20	1.10	0.85	0.90	0.73	0.80	0.88	6
7	6-7	0.73	1.25	1.05	0.90	0.85	1.00	0.77	0.77	0.95	7
8	7-8	0.73	0.95	1.00	0.80	0.75	1.00	0.72	0.80	0.85	8

Table S2. Depth profiles of Chl-*a* data (up to 50 m) measured at the sampling zones during 2018 and 2019 in the south Caspian Sea. Geographical location of the sampling zones is shown in the panel (b) of Fig. 1.

WINTER											
No.	Water depth (m)	Depth profile of Chl-*a* (µg/L) in different sampling zones									
		S1	S2	S3	S4	S5	S6	S7	S8	S9	S10
9	8-9	0.75	1.10	0.90	0.75	0.75	1.00	0.70	0.83	0.85	9
10	9-10	0.73	0.90	0.90	0.70	0.80	0.95	0.73	0.77		10
11	10-11	0.70	0.90	0.90	0.70	0.80	0.90	0.67	0.77		11
12	11-12	0.70	0.90	0.80	0.70	0.78	0.90	0.63	0.77		12
13	12-13	0.67	0.90	0.80	0.70	0.75	0.90	0.65	0.80		13
14	13-14	0.67	0.90	0.80	0.75	0.70	0.90	0.63	0.73		14
15	14-15	0.60	0.80	0.70	0.70	0.75	0.80	0.63	0.73		15
16	15-16	0.63	0.80	0.60	0.60	0.75	0.80	0.70	0.67		16
17	16-17	0.57	0.70	0.60	0.50	0.80	0.85	0.67	0.67		17
18	17-18	0.53	0.60	0.60	0.50	0.85	0.80	0.67	0.71		18
19	18-19	0.53	0.50	0.60	0.55	0.85	0.75	0.67	0.70		19
20	19-20	0.47	0.50	0.60		0.80	0.70	0.63	0.60		20
21	20-21	0.50	0.45	0.60		0.80	0.70	0.67	0.65		21
22	21-22	0.48	0.50	0.60		0.83	0.70	0.72	0.63		22
23	22-23	0.45	0.50	0.60		0.75	0.65	0.70	0.63		23
24	23-24	0.40	0.50	0.60		0.70	0.60	0.75	0.63		24
25	24-25	0.40	0.50	0.60		0.68	0.60	0.75	0.60		25
26	25-26	0.40	0.50	0.60		0.65	0.60	0.75	0.60		26
27	26-27	0.40	0.40	0.55		0.63	0.50	0.70	0.68		27
28	27-28	0.35	0.40	0.50		0.60	0.50	0.70	0.70		28
29	28-29	0.35	0.40	0.50		0.60	0.50	0.60	0.65		29
30	29-30	0.35	0.40			0.70	0.50	0.60	0.65		30
31	30-31	0.30	0.35			0.50	0.40	0.45	0.65		31
32	31-32	0.30	0.30			0.40	0.40	0.43	0.83		32
33	32-33	0.30	0.30			0.40	0.50	0.40	0.90		33
34	33-34	0.30	0.30			0.40	0.40	0.40	0.80		34
35	34-35	0.30	0.30			0.40	0.40	0.40	0.80		35
36	35-36	0.45	0.30			0.30	0.30	0.30	0.80		36

Table S2. Depth profiles of Chl-*a* data (up to 50 m) measured at the sampling zones during 2018 and 2019 in the south Caspian Sea. Geographical location of the sampling zones is shown in the panel (b) of Fig. 1.

WINTER											
No.	Water depth (m)	Depth profile of Chl-*a* (µg/L) in different sampling zones									
		S1	S2	S3	S4	S5	S6	S7	S8	S9	S10
37	36-37	0.40	0.30			0.30	0.30	0.30	0.70		37
38	37-38	0.35	0.30			0.30	0.25	0.30	0.70		38
39	38-39	0.50	0.30			0.30	0.30	0.30	0.70		39
40	39-40	0.30	0.30			0.30	0.30	0.30	0.70		40
41	40-41	0.40	0.30			0.30	0.20	0.30	0.70		41
42	41-42		0.30			0.30	0.20	0.30	0.70		42
43	42-43		0.30			0.30	0.20	0.30	0.70		43
44	43-44		0.30			0.30	0.20	0.30	0.80		44
45	44-45		0.35			0.30		0.30	0.90		45
46	45-46					0.30		0.30			46
47	46-47					0.30		0.30			47
48	47-48					0.30		0.30			48
49	48-49					0.30		0.30			49
50	49-50					0.25		0.25			50
AUTUMN											
No.	Water depth (m)	Depth profile of Chl-*a* (µg/L) in different sampling zones									
		S1	S2	S3	S4	S5	S6	S7	S8	S9	S10
1	0-1	0.64	2.73	2.30	2.60	1.82		0.82	0.48	0.15	1
2	1-2	0.65	2.90	2.40	2.40	1.36		1.01	0.81	0.23	2
3	2-3	1.52	2.65	2.30	2.60	1.76		1.21	1.05	0.28	3
	3-4	1.92	2.70	2.40	3.00	1.93		1.23	1.27	0.25	
5	4-5	1.30	2.90	2.40	2.90	1.70		1.22	1.48	0.32	5
6	5-6	1.48	2.30	2.90	2.80	1.60		1.15	1.55	0.40	6
7	6-7	1.47	2.00	2.30	2.70	4.32		1.09	1.63	0.40	7
8	7-8	1.65	1.95	2.50	2.80	1.73		1.13	1.67	0.40	8
9	8-9	1.55	1.80	2.60	2.40	1.45		1.11	1.60		9
10	9-10	1.67	2.10	2.55	2.30	1.40		1.10	1.53		10
11	10-11	1.58	1.95	2.20	2.35	1.50		1.05	1.55		11

Table S2. Depth profiles of Chl-*a* data (up to 50 m) measured at the sampling zones during 2018 and 2019 in the south Caspian Sea. Geographical location of the sampling zones is shown in the panel (b) of Fig. 1.

WINTER											
No.	Water depth (m)	Depth profile of Chl-*a* (µg/L) in different sampling zones									
		S1	S2	S3	S4	S5	S6	S7	S8	S9	S10
12	11-12	1.58	2.14	2.10	2.60	1.33		1.03	1.58		12
13	12-13	1.63	1.75	2.00	2.70	1.15		1.07	1.52		13
14	13-14	1.67	1.60	2.00	2.80	1.10		1.03	1.48		14
15	14-15	1.63	1.55	1.90	2.40	1.23		1.12	1.52		15
16	15-16	1.60	1.65	1.70	2.00	1.20		1.00	1.43		16
17	16-17	1.50	1.40	1.85	1.95	1.38	1.20	1.10	1.38		17
18	17-18	1.63	1.45	1.80	2.10	1.25	1.20	1.00	1.40		18
19	18-19	1.75	1.35	1.70		1.00	1.15	1.04	1.37		19
20	19-20	1.84	1.30	1.40		1.10	1.10	0.98	1.50		20
21	20-21	1.48	1.55	1.40		1.10	1.10	0.98	1.25		21
22	21-22	1.55	1.30	1.35		1.00	1.15	0.87	1.30		22
23	22-23	1.45	1.30	1.60		1.10	0.90	0.88	1.40		23
24	23-24	1.43	1.30	1.20		1.10	0.90	0.88	1.30		24
25	24-25	1.38	1.30	1.20		0.90	0.95	0.70	1.10		25
26	25-26	1.53	1.30			1.00	0.80	0.80	1.15		26
27	26-27	1.35	1.33			0.90	0.80	0.75	0.88		27
28	27-28	1.43	1.40			0.90	0.80	0.75	0.85		28
29	28-29	1.48	1.34			0.90	0.80	0.83	0.60		29
30	29-30	1.53	1.20			0.95	0.85	0.70	0.45		30
31	30-31	1.30	1.50			1.00	0.95	0.83	0.30		31
32	31-32	1.40	1.20			0.90	0.80	0.50	0.35		32
33	32-33	1.40	1.55			0.85	0.70	0.50	0.25		33
34	33-34	1.45	1.30			0.90	0.70	0.50	0.28		34
35	34-35	1.20	1.30			0.80	0.70	0.50	0.29		35
36	35-36	1.40	1.20			0.70	0.70	0.50	0.20		36
37	36-37	1.63	1.20			0.70		0.40	0.30		37
38	37-38		1.30			0.65		0.50			38
39	38-39		1.20			0.50		0.50			39

Table S2. Depth profiles of Chl-*a* data (up to 50 m) measured at the sampling zones during 2018 and 2019 in the south Caspian Sea. Geographical location of the sampling zones is shown in the panel (b) of Fig. 1.

WINTER											
No.	Water depth (m)	Depth profile of Chl-*a* (μg/L) in different sampling zones									
		S1	S2	S3	S4	S5	S6	S7	S8	S9	S10
40	39-40		1.25			0.40		0.40			40
41	40-41		1.10			0.70		0.40			41
42	41-42		1.25					0.40			42
43	42-43		1.25					0.45			43
44	43-44		1.20					0.40			44
45	44-45							0.40			45
46	45-46							0.40			46
